# Sublingual microcirculation does not reflect red blood cell transfusion thresholds in the intensive care unit—a prospective observational study in the intensive care unit

**DOI:** 10.1186/s13054-020-2728-7

**Published:** 2020-01-17

**Authors:** Jonas Scheuzger, Anna Zehnder, Vera Meier, Desirée Yeginsoy, Julian Flükiger, Martin Siegemund

**Affiliations:** Department for Anesthesia, Surgical Intensive Care Unit, Prehospital Emergency Medicine and Pain Therapy, University Hospital Basel, Petersgraben 4, 4031 Basel, Switzerland

**Keywords:** Sublingual microcirculation, Vessel perfusion, Capillary density, Transfusion, Critical illness, Intensive care unit

## Abstract

**Purpose:**

Hemoglobin (Hb) transfusion thresholds are established in intensive care units. A restrictive transfusion threshold (Hb 70–75 g/l) is recommended in septic patients, and a liberal transfusion threshold (Hb 90 g/l) for cardiogenic shock. It is unclear whether these historically adopted transfusion thresholds meet the challenges of individual patients.

**Methods:**

We evaluated microvascular flow index (MFI) and proportion of perfused vessels (PPV) in the sublingual microcirculation with CytoCam-IDF microscopy and near-infrared spectroscopy (NIRS). A study team-independent, treating intensivist assigned a total of 64 patients to 1 of 2 two transfusion thresholds, 43 patients to the Hb 75 g/l threshold and 21 patients to the Hb 90 g/l threshold, at a surgical intensive care unit. We performed microcirculatory measurements 1 h before and 1 h after transfusion of 1 unit of red blood cells.

**Results:**

Microcirculatory flow variables correlated negatively with pre-transfusion flow variables (ΔMFI: *ρ* = − 0.821, *p* <  0.001; ΔPPV: *ρ* = − 0.778, *p* <  0.001). Patients with good initial microcirculation (cutoffs: MFI > 2.84, PPV > 88%) showed a deteriorated microcirculation after red blood cell transfusion. An impaired microcirculation improved after transfusion. At both transfusion thresholds, approximately one third of the patients showed an initially impaired microcirculation. In contrast, one third in every group had good microcirculation above the cutoff variables and did not profit from the transfusion.

**Conclusion:**

The data suggest that the established transfusion thresholds and other hemodynamic variables do not reflect microcirculatory perfusion of patients. Blood transfusion at both thresholds 75 g/l and 90 g/l hemoglobin can either improve or harm the microcirculatory blood flow, questioning the concept of arbitrary transfusion thresholds.

## Background

Transfusion of red blood cells (RBC) is associated with various risks and complications. Allergic and immune transfusion reactions, hyperkalemia due to potassium released by the RBC, infections from transfusion-transmitted pathogens, and volume and iron overload can occur. Therefore, RBC transfusions should be clearly indicated and carefully evaluated. Until now, no functional monitoring of the effectiveness of RBC transfusions has been established.

Today, clinicians rely on transfusion thresholds based on hemoglobin (Hb) level to initiate RBC transfusion. For years, there was an ongoing debate whether transfusion thresholds should generally be shifted from a liberal (Hb 90–100 g/l) to a restrictive transfusion threshold (Hb 70–75 g/l). In a meta-analysis from 2018 including 19,000 intensive care patients, Carson et al. [1] found that restrictive versus liberal transfusion thresholds resulted in no difference in 30-day mortality, recovery rate, and myocardial infarction. However, in a meta-analysis of 17 randomized controlled trials, Fominskiy et al. [2] observed a decreased mortality rate among patients assigned to a liberal transfusion strategy during the perioperative period. Murphy et al. [3] found that a restrictive transfusion threshold after cardiac surgery was not superior to a liberal threshold. Likewise, a meta-analysis [4] from 2016 found that a restrictive transfusion threshold might affect the outcome of cardiovascular and elderly orthopedic patients. Despite these uncertainties, most up-to-date guidelines recommend a restrictive transfusion protocol for all patients except for those with active bleeding or untreated cardiovascular diseases [5–7]. Overall, these studies used negative outcome but not possible benefits for the patients to delimit the current transfusion thresholds. Whether transfusion thresholds can be seen as arbitrary and should, therefore, be individualized is a matter of discussion [8, 9].

Organ perfusion and tissue oxygenation take place by direct exchange of oxygen from erythrocytes to the endothelial surface of small vessels and capillaries of the microcirculatory system. Previous studies in septic [10] and cardiac [11] patients have demonstrated that persistent microcirculatory flow (MCF) alterations unresponsive to therapy are independently associated with adverse outcome. Despite intact macro-hemodynamic parameters (e.g., blood pressure and cardiac output), tissue hypoxia due to MCF collapse or dysregulation occurs in shock patients [12, 13]. Investigation of human MCF on tissue surface after transfusion is now possible with the latest generation of microcirculation microscopes.

In this study, we evaluated the influence of RBC transfusion on the microcirculation at the two widely accepted transfusion thresholds (TTHs; 75 g/l and 90 g/l) and tested the hypothesis that RBC transfusion improves microcirculation independent of the accepted transfusion thresholds.

## Materials and methods

### Patients

After receiving approval from the local ethics committee (Ethics Committee of Northwestern and Central Switzerland, EKNZ, project ID: 2017-01190), this single-center observational study was performed in the 22-bed surgical intensive care unit at the University Hospital of Basel. We included patients with various medical conditions such as trauma, sepsis, postoperative bleeding, or cardiogenic shock receiving a RBC transfusion from September 2017 to September 2018. The TTH of 75 g/l or 90 g/l was set prior to inclusion by the treating intensivist independent of the study. We excluded patients aged < 18 years, patients requiring mechanical assist devices, and patients with orofacial trauma, active oral bleeding, or any other condition complicating sublingual microcirculatory measurement.

### Protocol

We performed sublingual microcirculatory measurements within 1 h before (*T*_1_) and within 1 h after (*T*_2_) transfusion of one 200 ml unit of leukocyte-depleted RBC. Measurements were planned and initiated upon the first order of one or more units of RBC and a pre-assignment to a TTH of 75 g/l or 90 g/l Hb during ICU admission.

The intensivist in charge ordered RBC based on the TTH and his/her clinical experience. We recorded hemodynamic measurements of the fully monitored patients (i.e., mean, systolic, and diastolic arterial pressures (MAP, SAP, and DAP), Hb concentration, and SpO_2_ (peripheral oxygen saturation)) at *T*_1_ and *T*_2_. In addition, the sequential organ failure assessment (SOFA) score was recorded at *T*_1_*.*

Lactate levels were measured regularly before and after RBC transfusion. The normal range of lactate is from 0.5 to 1.5 mmol/l and is considered as elevated at 2 mmol/l. We chose 1.8 mmol/l as the threshold, as it lies between the normal and elevated range. The in-house laboratory reports lactate levels above 1.8 mmol/l to the physician in charge. Data were also collected from regional oxygen saturation (rSO_2_) based on near-infrared spectroscopy (NIRS) using a SenSmart™ (Model X-100 M, Nonin, Plymouth, MN, USA) universal oximeter. We placed the sensors over the thenar prominence, the anterior region of the foot, and the frontotemporal region of the brain. After 5 min of measurement, we recorded all tissue saturations from the same side of the body at *T*_1_ and *T*_2_.

### Microcirculation

We used the latest microcirculation camera, CytoCam (Braedius, Netherlands), based on incident dark-field illumination technology. This handheld microscope currently provides the best optical resolution and is suitable for bedside use [14].

The investigator (JS), who had attended a microcirculation-training course and had performed microcirculatory measurements regularly before the study, acquired all videos. He recorded a minimum of five videos from different sublingual areas for each time point (*T*_1_ and *T*_2_). We selected the three best videos according to the microcirculatory quality score (MIQS) [15], following the recommendation of the second consensus for microcirculation assessment [16].

The tip-like microscope was carefully applied in the sublingual fold to avoid pressure artifacts. We recorded videos 175 frames in length at a rate of 25 frames per second. The videos were then selected, cropped, and stabilized with CytoCam Tools version 1.7.12 (Braedius, Netherlands). We applied stricter rules regarding stabilization than required by the MIQS and cropped the videos with a movement no larger than one quarter of the frame length. Most videos were cropped to a video length of 3–5 s.

The investigator and a blinded independent observer performed offline analysis. Time point and order of the videos were blinded for offline analysis. Microvascular flow index (MFI) was assessed by scoring the mean of the predominant flow type (0 = no flow, 1 = intermitted flow, 2 = sluggish flow, and 3 = continuous flow) of the four quadrants of the video screen. Perfused vessel density (PVD), an estimate of functional capillary density, and the proportion of perfused vessels (PPV) were calculated as percentage and number of crossings of perfused vessels per total length of a grid of three equidistant horizontal and vertical lines (De Backer Score) [17].

According to the developer of the CytoCam, we calculated with the total grid length of 7.9825 mm (3 × 1.1536 mm + 3 × 1.5456 mm). Since automatic software analysis provided by CytoCam is only comparable with automatic vascular analysis for total vessel density (TVD) but not for PPV and PVD, we deemed offline analysis according to De Backer to be most suitable for clinical practice.

We analyzed 354 videos. Of every measurement, we calculated the mean of the three corresponding videos of each time point and patient for the study calculation.

### Statistical analysis

To test whether the incidence of patients with an MFI > 2.5 in the TTH 90 g/l group is larger than 2%, we calculated the 95% confidence interval (CI) around the observed incidence and concluded the incidence to be clearly larger than 2% if the entire 95% CI lies above 2% [18]. We aimed for an 80% power to find a 95% CI that lies entirely above 2%. Assuming a true incidence of 15%, a sample size of 20 is required to achieve this power [19].

We estimate a 2% incidence for RBC transfusion despite intact MFI > 2.5 as relevant, based on the total number of RCB transfusions worldwide. It has been estimated that about 40% of patients receive one or more RBC transfusions while in the ICU. We expected the prevalence of transfusion to occur in about one third and two thirds of patients in the TTH 90 g/l and TTH 75 g/l groups, respectively [20, 21]. To reach a sample size of 20 patients in the TTH 90 g/l group, we planned to include a total of 60 patients, 40 of whom would be enrolled in the TTH 75 g/l group.

Further, we calculated the correlation between lactate decrease and the increase in hemodynamic variables and Hb after transfusion with the change in MCF (MFI, TVT, PPV, and PVD) and tested whether it differs from zero. For all tests, alpha (*p* > 0.05) was considered significant. Alpha was not corrected regarding the problem of multiple testing, indicating that the combined alpha level for the two primary tests is about 10%.

Calculations were performed using R Studio, version 1.1.423 (R Studio, Inc., Boston, MA, USA, 2009–2018). For continuous variables, data are presented as median and interquartile range. We compared variables before and after blood transfusion in the TTH 75 g/l group and TTH 90 g/l group using the paired Wilcoxon signed-rank test for non-normally distributed values. Correlations between variables were investigated using Spearman’s *ρ*.

To describe interrater reliability of the offline analysis (MFI, counted number of vessels with flow, and counted number of vessels with no or intermitted flow), we used a two-way random-effects consistency model for the intra-class correlation coefficient (ICC). Results of the ICC are presented as ICC with boundaries of the 95% CI and *F*-tested significance level.

## Results

Characteristics of the 64 patients enrolled in the study are listed in Table 1. Most patients were in hemorrhagic shock (34%) before transfusion. Twenty-nine patients (45%) were intubated. Of the patients assigned to the TTH 90 g/l group, 57% had cardiogenic shock. More patients in the TTH 90 g/l group had a lactate concentration > 1.8 (43% vs. 21%, respectively) and were mechanically ventilated (62% vs. 37%, respectively) than in the TTH 75 g/l group. Eight patients (13%) died during ICU stay.

**Table 1:** Patient characteristics

Characteristics	Total (*n* = 64)	Hb 75 g/l (*n* = 43)	Hb 90 g/l (*n* = 21)
Male, *n* (%)	39 (61)	26 (60)	13 (61)
Age, years	68 (58–77)	67 (53–78)	70 (64–77)
SOFA	7 (4–11)	5 (3–8)	10 (7–12)
Septic shock, *n* (%)	12 (19)	11 (26)	2 (10)
Cardiogenic shock, *n* (%)	21 (33)	9 (21)	12 (57)
Hemorrhagic shock, *n* (%)	22 (34)	15 (35)	6 (29)
Other, *n* (%)	9 (14)	8 (19)	1 (5)
Intubation, *n* (%)	29 (45)	16 (37)	13 (62)
Hb before RBCT, (g/l)	75 (72–81)	73 (71–75)	85 (81–88)
Lactate > 1.8 (mmol/l) before RBCT, *n* (%)	18 (28)	9 (21)	9 (43)
Lactate before RBCT (mmol/l)	1.2 (0.8–2.0)	1.1 (0.8–1.7)	1.6 (1.1–2.5)
NOR (μg/kg/min)	0.03 (0.00–0.08)	0.00 (0.00–0.04)	0.05 (0.03–0.07)
SpO_2_ before RBCT	98 (95.5–100)	99 (96–100)	97 (95–100)
Days to RCBT	3 (2–6)	3 (1–4)	4 (2–7)
Total days in ICU	4.5 (2–11)	4 (2–11)	7 (4–11)
Death in ICU, *n* (%)	8 (13)	6 (14)	2 (10)

*Hb* hemoglobin, *SOFA* sequential organ failure assessment, *RBCT* red blood cell transfusion, *NOR* noradrenaline, *SpO*_*2*_ peripheral oxygen saturation, *ICU* intensive care unit

We recorded over 600 videos, 354 of which fulfilled the MIQS criteria for offline analysis. We recorded 64 patients before and 55 after transfusion. We lost 9 patients due to delayed transfusion protocol and emergency procedures. The median MIQS of the video recordings was 2 (1–2) with a maximum of 5. Median time after completed RBC transfusion and the recording at *T*_2_ was 18 (10–30) min. The ICC for MFI for the raters was 0.86 (0.83–0.88, *p* < 0.001), for counts of normal flow vessels in the grid 0.85 (0.82–0.89, *p* < 0.001), and for counts of vessels with no/intermediate flow 0.92 (0.91–0.94, *p* < 0.001). These results indicate a good interrater reliability [22].

### Microcirculation

Microcirculation variables are presented in Table 2. A clearly impaired microcirculation indicated by a mean MFI < 2.5 (based on three recordings) was found in the TTH 75 g/l group (*n* = 16, 37%) and in the TTH 90 g/l group (*n* = 6, 29%) before transfusion. MFI, PPV, TVD, and PVD increased significantly from before to after one RBC transfusion in patients in the TTH 75 g/l group or in septic patients, but not in the TTH 90 g/l group (Table 3).

**Table 2:** Number and percentage of flow indices at different cutoff levels

	Total (*n*)		MFI < 2.5, *n* (%)		MFI ≥ 2.84, *n* (%)	PPV ≥ 88, *n* (%)
	Before RBCT	After RBCT	Before RBCT	After RBCT	Before RBCT	Before RBCT
All	64	55	22 (34)	6 (11)	21 (32)	22 (34)
TTH 75 g/l	43	39	16 (37)	3 (8)	13 (30)	16 (37)
TTH 90 g/l	21	16	6 (29)	3 (20)	8 (38)	6 (29)
SENS/SPEC					0.66/0.91	0.64/0.82
PpV/NpV					0.91/0.85	0.64/0.83

*RBCT* red blood cell transfusion, *MFI* microvascular flow index, *PPV* proportion of perfused vessels, *TTH* transfusion threshold, *SENS* sensitivity, *SPEC* specificity, *PpV* positive predictive value, *NpV* negative predictive value

**Table 3:** Changes in microvascular flow index and proportion of perfused vessels before and after RBC transfusion

	MFI, median (IQR)		*p* value	PPV, median (IQR)		*p* value	TVD, median (IQR)		*p* value	PVD, median (IQR)		*p* value
	Before RBCT	After RBCT		Before RBCT	After RBCT		Before RBCT	After RBCT		Before RBCT	After RBCT	
All	2.75 (2.33–2.91)	2.83 (2.60–2.91)	0.002	83.3 (70.5–89.7)	85.3 (78.4–90.0)	0.001	10.38 (9.51–11.62)	11.27 (10.23–12.45)	0.005	8.68 (6.95–9.74)	9.60 (8.35–10.46)	< 0.001
Septic	2.41 (2.00–2.77)	2.91 (2.91–3.00)	0.008	71.7 (66.3–85.3)	89.2 (86.4–92.6)	0.001						
TTH 75 g/l	2.70 (2.33–2.91)	2.83 (2.64–2.91)	< 0.001	82.8 (67.9–89.6)	85.3 (79–89.5)	< 0.001						
TTH 90 g/l	2.75 (2.41–2.62)	2.75 (2.5–2.91)	0.665	82.7 (71.6–88.4)	86.3 (74.5–91.3)	0.308						

*RBCT* red blood cell transfusion, *MFI* microvascular flow index, *IQR* interquartile range, *PPV* proportion of perfused vessels, *TVD* total vessel density, *PVD* perfused vessel density, *TTH* transfusion threshold

**Fig. 1:**
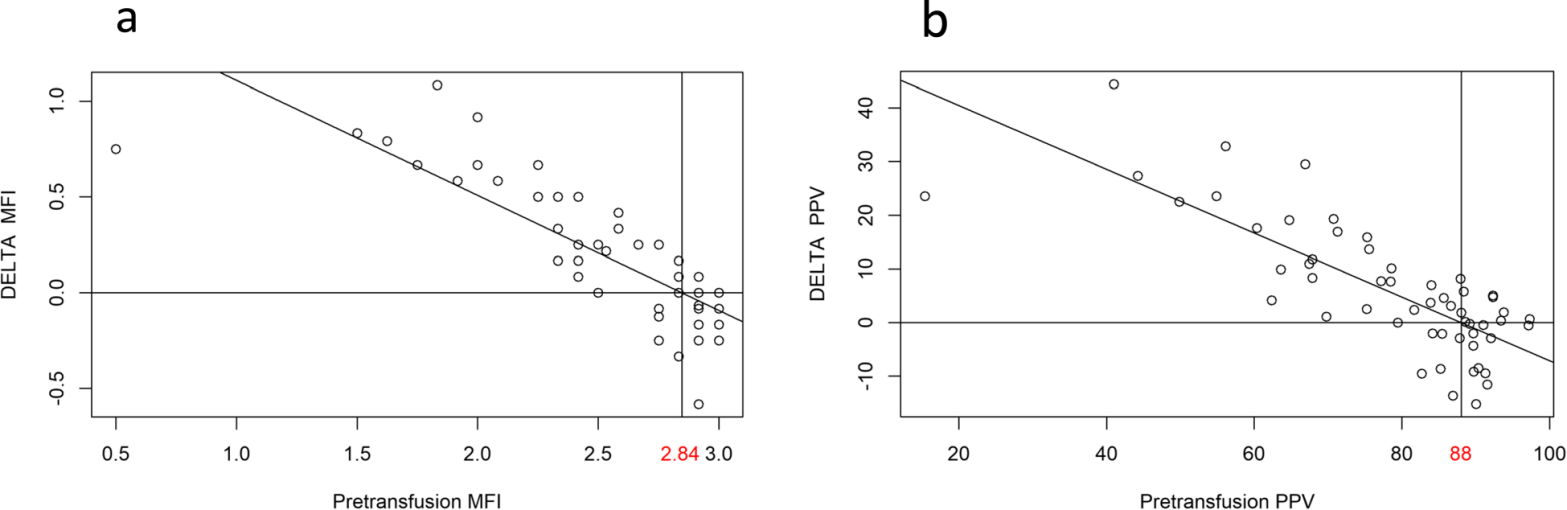
ΔMFI (**a**) and ΔPPV (**b**) after RBC transfusion in correlation with the pre-transfusion baseline. MFI, microvascular flow index; RBC, red blood cell; PPV, proportion of perfused vessels

Overall change in flow variables before and after transfusion correlated negatively with pre-transfusion MFI, PPV, TVD, and PVD with a ΔMFI (*ρ* = − 0.821, *p* < 0.001), ΔPPV (*ρ* = − 0.778, *p* < 0.001) (Fig. 1), ΔTVD (*ρ* = − 0.402, *p* = 0.002), and ΔPVD (*ρ* = − 0.595, *p* < 0.001) (Additional file 2: Figure S2). This overall negative correlation was also found in the TTH 75 g/l group with ΔMFI (*ρ* = − 0.814, *p* < 0.001) and ΔPPV (*ρ* = − 0.822, *p* < 0.001), and in the TTH 90 g/l group with ΔMFI (*ρ* = − 0.647, *p* = 0.007) and ΔPPV (*ρ* = − 0.570, *p* = 0.023).

A linear regression model produced cutoff levels for improvement in MCF after RBC transfusion for MFI at 2.84 and for PPV at 88.4% (Fig. 1). Pre-transfusion microcirculation exceeding these cutoff levels was most likely to deteriorate after the RBC transfusion. Thirty percent and 37% of patients in the TTH 75 group, and 38% and 29% in the TTH 90 group had pre-transfusion MFI and PPV above cutoff levels, respectively. For both cutoff levels, specificity was higher than sensitivity (Table 2). We re-evaluated all results after the exclusion of one outlier with extreme deterioration of MCF (Fig. 1) to detect and avoid a possible distortion of the linear regression model. The cutoff level for PPV decreased to 87% and for MFI to a value of 2.81. No change according to any of the significance levels was observed.

Circulatory parameters and NIRS values are presented in Table 4. Hb increased significantly after RBC transfusion within the expected range of 10 to 15 g/l in both TTH groups. We found no correlation with pre-transfusion Hb and ΔMFI (*ρ* = − 0.242), ΔPPV (*ρ* = − 0.187), ΔTVD (*ρ* = − 0.161), and ΔPVD (*ρ* = − 0.124). Changes in MAP, SAP, and DAP were not correlated with ΔMFI and ΔPPV (Additional file 3: Table S1). Lactate levels did not significantly decrease within 2 h after RBC transfusion (*p* = 0.82) but were significantly lower after 8 h (*p* = 0.009). No correlation was found in the change in lactate levels, ΔMFI (*ρ* = 0.120, *p* = 0.503), and ΔPPV (*ρ* = 0.056, *p* = 0.752) up to 10 h after RBC transfusion.

**Table 4:** Circulatory parameters and near-infrared spectroscopy

Values	Before transfusion	After transfusion	*p* value
Hb, g/l	75 (71–81)	87 (82–93)	< 0.001
MAP	72 (63–79)	75 (67–81)	0.15
SAP	111 (94–130)	111 (100–128)	0.55
DAP	52 (48–62)	55 (50–64)	0.20
SpO_2_	98 (96–100)	98 (96–100)	0.75
rSO_2_ (palm)	65 (62–68)	66 (62–69)	0.309
rSO_2_ (foot)	56 (53–60)	58 (52–65)	0.124
rSO_2_ (brain)	67 (62–70)	68 (65–72)	< 0.001

*Hb* hemoglobin, *MAP* mean arterial pressure, *SAP* systolic arterial pressure, *DAP* diastolic arterial pressure, *rSO*_*2*_ regional oxygen saturation

**Fig. 2:**
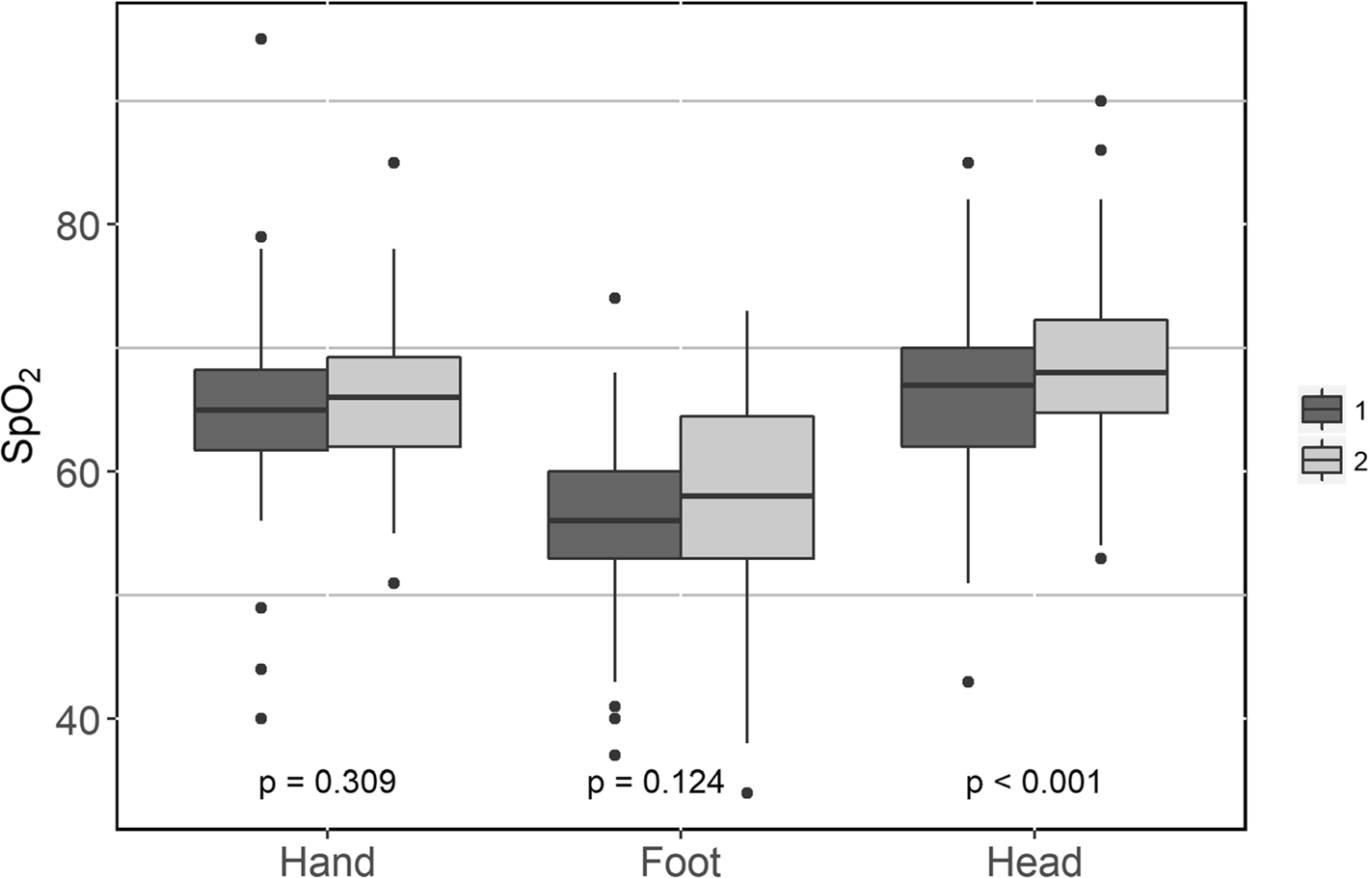
Tissue oxygenation before and after one RBC transfusion (boxplot). RBC, red blood cell

Tissue oxygenation measured by NIRS increased significantly in the frontotemporal region, but not in the extremities, after RBC transfusion (Fig. 2). Pre-transfusion MFI and PPV did not correlate with pre-transfusion tissue oxygen saturations (Additional file 4: Table S2).

## Discussion

Our study shows that the intention to increase oxygen transport capacity by infusion of one unit of RBC may have both positive and negative effects on the microcirculation of critically ill patients at both existing TTH levels. Pivotal for this effect is the quality of pre-transfusion MCF. We were able to define a cutoff value for pre-transfusion MFI of 2.84 and a pre-transfusion PPV of 88%. Transfusion of one unit of RBC above these cutoff values did not improve microcirculation. On the contrary, transfusion impaired MCF in patients with a good baseline microcirculation. To our knowledge, this is the first study to focus on the two TTHs (i.e., 75 g/l Hb and 90 g/l Hb) commonly used in the ICU with regard to sublingual microcirculation. With 354 evaluated videos of sublingual microcirculation from 64 ICU patients, to date, this is the largest study to observe the effects of RBC transfusion on microcirculation in critically ill patients at the bedside.

More patients showed impaired microcirculation (MFI < 2.5) in the TTH 75 g/l group than in the TTH 90 g/l group (37% vs. 29%, respectively). Microcirculation in these patients responded favorably to RBC transfusion. However, the same percentage of patients in both the TTH 75 g/l and TTH 90 g/l groups (37% and 29%, respectively) had a baseline PPV > 88%, representing a very good initial sublingual microcirculation. Interestingly, microcirculation in these patients was negatively affected by the RBC transfusion. With regard to microcirculation, only one third of the RBC transfusions at the given thresholds appeared to be indicated for improvement of MCF. Thus, transfusion exclusively based on a predefined TTH and, therefore, tolerating impaired MCF in extreme anemia might be harmful and should be scrutinized.

The current transfusion thresholds were defined because several studies with Hb thresholds of 70–75 g/l and 90–100 g/l in different patient cohorts showed non-inferiority of the lower boundary with regard to mortality. However, increased mortality with lower Hb levels has been detected in patients who declined RBC transfusion [23], and therefore, it is unclear whether an even lower TTH in an individual patient (e.g., younger adults with normal heart function) may be beneficial, or if special patient groups would benefit from higher thresholds. Therefore, future studies should aim to evaluate the benefits of RBC transfusions in patients with clear signs of an impaired microcirculation. Determination of arbitrary transfusion thresholds may harm as many patients as it will help.

In a study of 30 hemodynamically stable trauma patients, Weinberg et al. [24] described a negative correlation between RBC transfusion and baseline sublingual microcirculation. They transfused patients with similar clinical appearance in a Hb range of 60–70 g/l and observed that patients with impaired pre-transfusion sublingual microcirculation responded favorably to a single RBC transfusion. In 24 perioperative patients undergoing cardiac surgery, Yuruk et al. [25] described an improvement in sublingual microcirculatory density after 1 RBC transfusion. Two studies in septic patients, performed in 2007 with 35 patients [26] and in 2011 with 21 patients [27], did not show impressive alterations of the sublingual microcirculation after one RBC transfusion. However, the earlier study detected a significantly lower baseline flow in patients who experienced an increase in capillary perfusion > 8% [26]. Furthermore, Sadaka et al. measured 11 out of 21 patients using side-stream dark-field microscopy [27]. In these 11 patients, in-hospital mortality was 45%, and the authors reported a very low baseline MFI of < 2 in 9 of their patients. However, considerable variability was detected among the patients. Pranskunas et al. [28] observed a significant improvement of MFI after blood and fluid administration in 50 ICU patients depending on a baseline MFI < 2.6. In this study, 66% of the patients had an MFI ≤ 2.6, a doubled incidence compared to our results although they found a similar median PPV of 88%. One reason for this discrepancy may be that our study applied stricter criteria for impaired microcirculation with 3 out of 4 MFI readings < 2.5. A point-in-time assessment of 1 sublingual microcirculation measurement in 501 randomly selected patients without intervention in 36 ICUs found an abnormal MFI of < 2.6 in only 17% of patients [29]. However, this study focused on the comparison of a large heterogeneous ICU group rather than on the incidence of MCF abnormalities, presumably resulting in an underestimation of the true incidence of microcirculatory dysfunction. In 2017, Tanaka et al. [30] demonstrated improved sublingual microcirculation (using side-stream dark-field microscopy) after RBC transfusion independent of circulatory parameters in 15 hemorrhagic shock patients. In agreement with our findings, Tanaka et al. detected a negative correlation between improved microcirculation after transfusion compared to pre-transfusion. Further, the authors also suggested that focusing exclusively on Hb concentration as a transfusion trigger could be detrimental in some cases.

The 2013 Transfusion Requirements in Septic Shock (TRISS) trial [31] compared sepsis patients with TTH 70 g/l versus TTH 90 g/l and found no difference in 60-day mortality. Concerning our findings, where approximately one third in each TTH group benefited from the transfusion and another two thirds in each group had no clear benefit or deteriorated, it is reasonable to presume that the effect of transfusion on mortality in the TRISS trial went undetected. Interindividual response to RBC transfusion may be masked by the fact that positive and negative microcirculatory responses occurred at both threshold levels. Therefore, a further distinction of outcome based solely on Hb levels seems erratic.

The results of the TRISS study contributed to the most recent guidelines of the 2017 Surviving Sepsis Campaign [6]. In agreement with other organizations (e.g., the American Association of Blood Banks), these guidelines recommend RBC transfusion in septic patients with a Hb TTH below 70 g/l. Myocardial ischemia, severe hypoxia, and acute hemorrhage allow a higher threshold. Further, these guidelines emphasize the incorporation of individual patient characteristics and conditions and that transfusions should not be based on Hb levels alone. Nevertheless, except for Hb thresholds, clinicians lack a definite indication for RBC transfusion. Several studies have reported impaired microcirculation to be more severe in non-survivors of sepsis, to be associated with organ failure and death, and to be a sensitive predictor of outcome [32–35]. Despite other findings [36], we found septic patients to be responsive to RBCT. Measurement of sublingual microcirculation may provide additional information for clinicians in the assessment of critically ill patients and may serve as a guide for RBC transfusion, even above the current threshold of 70 g/l (e.g., in patients with septic cardiomyopathy or after initial fluid resuscitation).

In patients with intact baseline microcirculation, we were able to detect a deterioration in microcirculation after RBC transfusion. The reason for this is not yet fully understood. Aggregability and deformability of the transfused RBC may play a role. Under stable microcirculatory conditions, RBC transfusion may disturb the fragile balance between fluids, flow, and erythrocytes. Non-functional RBC and cell components can aggravate aggregability and, therefore, reduce MCF. In 2014, Donati et al. [37] described higher flow velocity and PPV in patients with leukodepleted RBCT versus non-leukodepleted RBCT. We think the immune response plays an important role regarding the efficacy of RBC. Despite human leukocyte antigen (HLA) compatibility, homologous RBC transfusions trigger the immune system of the recipient and may lead to destabilization of the microcirculation in critically ill patients.

The prevalence of MCF alterations is variable over time in patients with shock. Extended fluid resuscitation and treatment of the underlying cause of the shock type may restore impaired microcirculation. We chose to include patients early after admission (mean 3 days) to detect impaired microcirculation in the early stages of shock. Tachon et al. [38] detected microcirculatory alterations in traumatic hemorrhagic shock lasting for 72 h despite restoration of the microcirculation. With the narrow time frame of examination before and after RBC transfusion, change of MCF due other interventions during this time period is minimized.

Although we measured a significant increase in tissue oxygenation in the frontotemporal region of the brain after RBC transfusion, we found no correlation with pre-transfusion microcirculatory variables. It is possible that the frontotemporal region, as a part of the brain circulation, benefits more from RBC transfusion in patients with shock. More likely, NIRS with its relatively high catchment area responds to the increased oxygen transport capacity. As we only measured tissue oxygenation within 1 h after RBC transfusion, an increase in tissue oxygenation in the extremities might only be visible later. Nevertheless, 80% of the patients with a low pre-transfusion MFI showed an increase in frontotemporal tissue oxygenation (Additional file 1: Figure S1).

In accordance with other studies, hemodynamic variables remained unaffected by RBC transfusion [26, 28, 30, 39] and, therefore, should not be used as an indicator for transfusion.

The current study has some limitations that should be noted. First, the study was not powered to evaluate outcome. Although impaired microcirculation predicts outcome, we do not know if the changes in MCF after a single RBC transfusion influence outcome. More, the two point-in-time measurements do not reflect other alterations of MCF over the full duration of shock. A single-center study will not be able to recruit enough patients for such an outcome. Our idea was to generate a hypothesis for a larger study to compare microcirculation-guided RBC transfusion in contrast to Hb threshold alone. Second, because transfusion threshold was assigned by the physician in charge and not performed randomly, selection bias cannot be excluded. Third, we lost five patients in the TTH 90 group due to immediate surgical intervention. Four of these patients clearly had an impaired pre-transfusion microcirculation (mean MFI < 2.6). This may be one reason why we were not able to detect a significant increase in microcirculation in this group after the RBC transfusion. Fourth, microcirculation variables were evaluated manually and not using an automatic software analysis (AVA). AVA software is very time-consuming and is not suitable for a bedside clinical approach. Software analysis provided by CytoCam is fast, but except for TVD, microcirculatory variables did not correlate well with the AVA and the De Backer Score [40]. To use microcirculatory perfusion clinically in the context of RBC transfusion, new and fast bedside analysis techniques are mandatory and have been developed recently [41].

## Conclusion

In this study, only one third of patients assigned to either transfusion threshold 75 g/l or 90 g/l Hb showed an apparently impaired microcirculation (mean MFI < 2.5, considering all three videos per patient). Approximately, the same proportion of patients in each group showed an intact microcirculation (cutoff PPV of 88%) and a decrease in MCF after RBC transfusion. The MCF of the remaining patients was only mildly affected by the RBC transfusion. Altered MCF after RBC transfusion correlated negatively with baseline values (Fig. 1). This implies that an increase of oxygen transport capacity by RBC transfusion has the potential to either increase or decrease the oxygen availability in the microcirculation depending on pre-transfusion microcirculatory conditions. Conventional transfusion thresholds and additional hemodynamic variables alone or in combination did not predict microcirculatory response and may be complemented by sublingual microcirculatory monitoring in the future.

## Supplementary information

**Additional file 1: Figure S1.** Change in tissue oxygenation over frontotemporal region of brain, after RBC transfusion in patients with impaired microcirculation at baseline (MFI < 2.5). *RBC* = red blood cell; *MFI* = microvascular flow index.

**Additional file 2: Figure S2.** Δ TVD (A) and Δ PVD (B) after RBC transfusion in correlation with the pre-transfusion baseline. *TVD* = total vessel density; *RBC* = red blood cell; *PVD* = perfused vessel density.

**Additional file 3: Table S1.** Correlations of microvascular variables and hemodynamic variables.

**Additional file 4: Table S2.** Correlations of tissue oxygenation and pre-transfusion MFI/PPV.

## Data Availability

The data that support the findings of this study are available from the corresponding author upon reasonable request.

## Abbreviations

AVA: Automatic software analysis
CI: Confidence interval
DAP: Diastolic arterial pressure
Hb: Hemoglobin
HLA: Human leukocyte antigen
ICC: Intra-class correlation coefficient
MAP: Mean arterial pressure
MCF: Microcirculatory flow
MFI: Microvascular flow index
MIQS: Microcirculatory quality score
NIRS: Near-infrared spectroscopy
PPV: Proportion of perfused vessels
PVD: Perfused vessel density
RBC: Red blood cells
rSO_2_: Regional oxygen saturation
SAP: Systolic arterial pressure
SOFA: Sequential organ failure assessment
SpO_2_: Peripheral oxygen saturation
*T*_1_: Time point 1
*T*_2_: Time point 2
TRISS: Transfusion Requirements in Septic Shock
TTH: Transfusion threshold
TVD: Total vessel density
