# Supplementary figures and images for: Sublingual microcirculation does not reflect red blood cell transfusion thresholds in the intensive care unit—a prospective observational study in the intensive care unit
Source: Crit Care. 2020 Jan 17;24:18. doi: 10.1186/s13054-020-2728-7 (PMC6969438; doi:10.1186/s13054-020-2728-7)

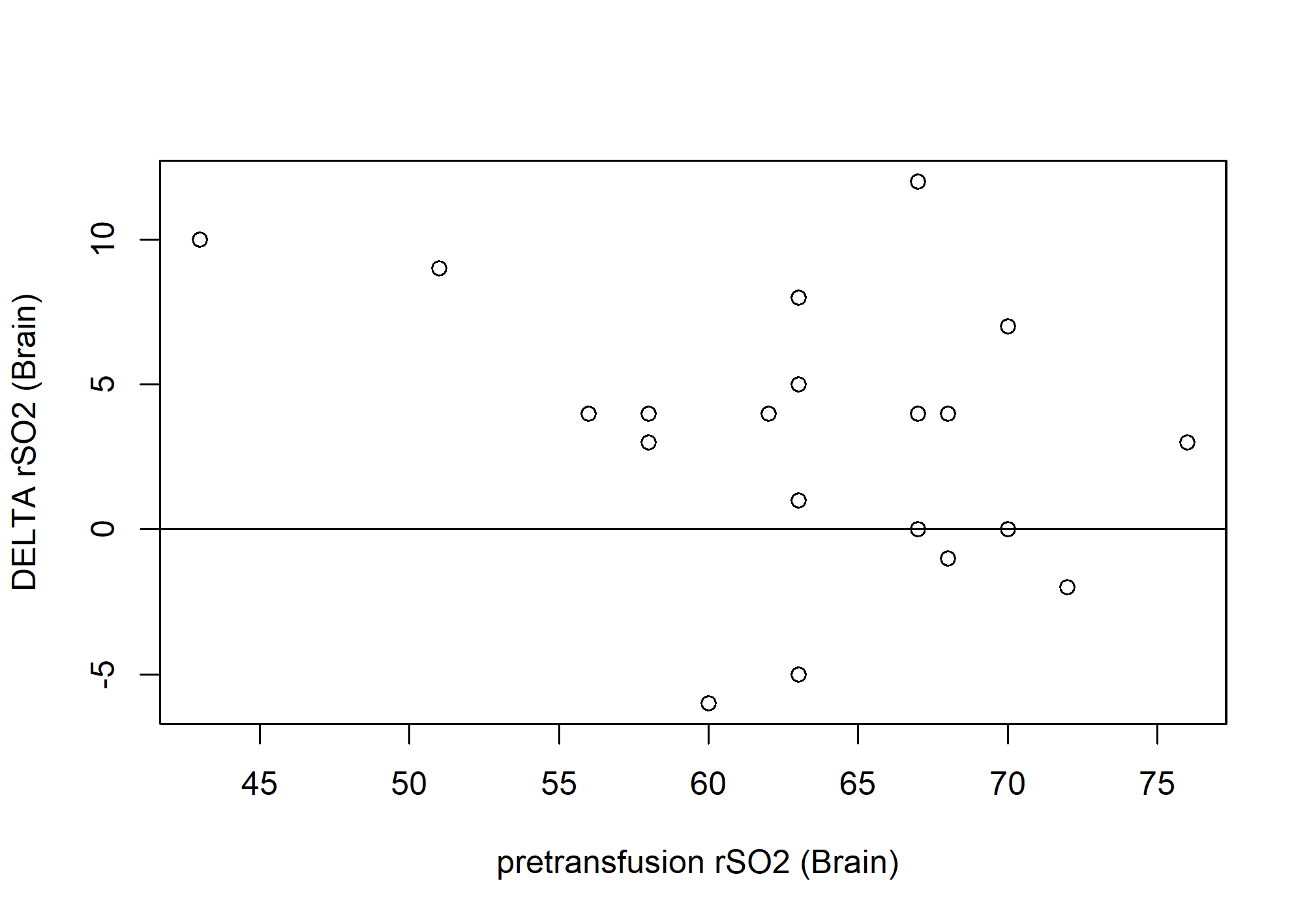


DELTA rSpO2 (Brain)

Pretransfusion rSpO2 (Brain)

Supplement: Supplementary file 1 — Additional file 1: Figure S1. Change in tissue oxygenation over frontotemporal region of brain, after RBC transfusion in patients with impaired microcirculation at baseline (MFI < 2.5). RBC = red blood cell; MFI = microvascular flow index. [file 13054_2020_2728_MOESM1_ESM.docx]

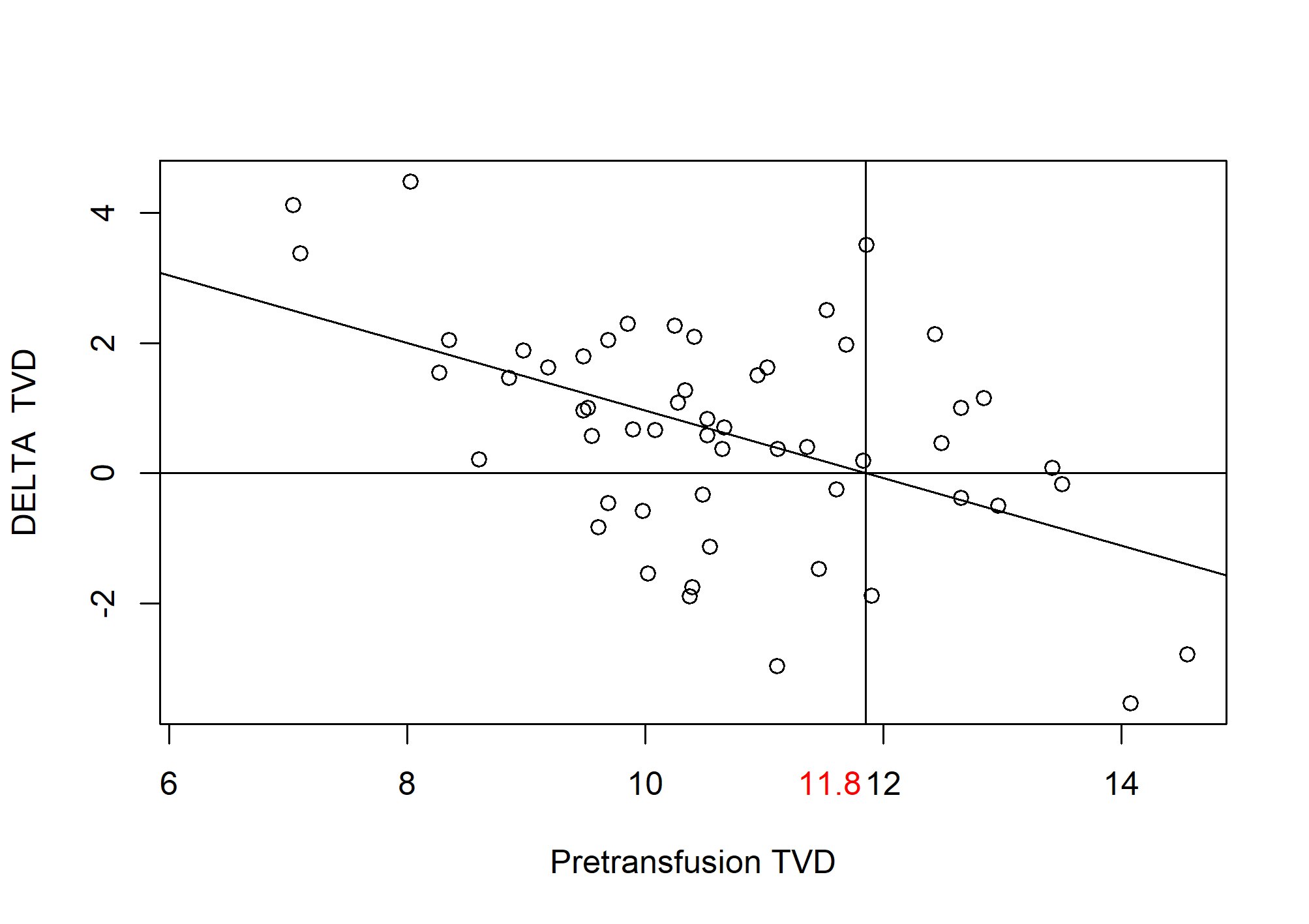

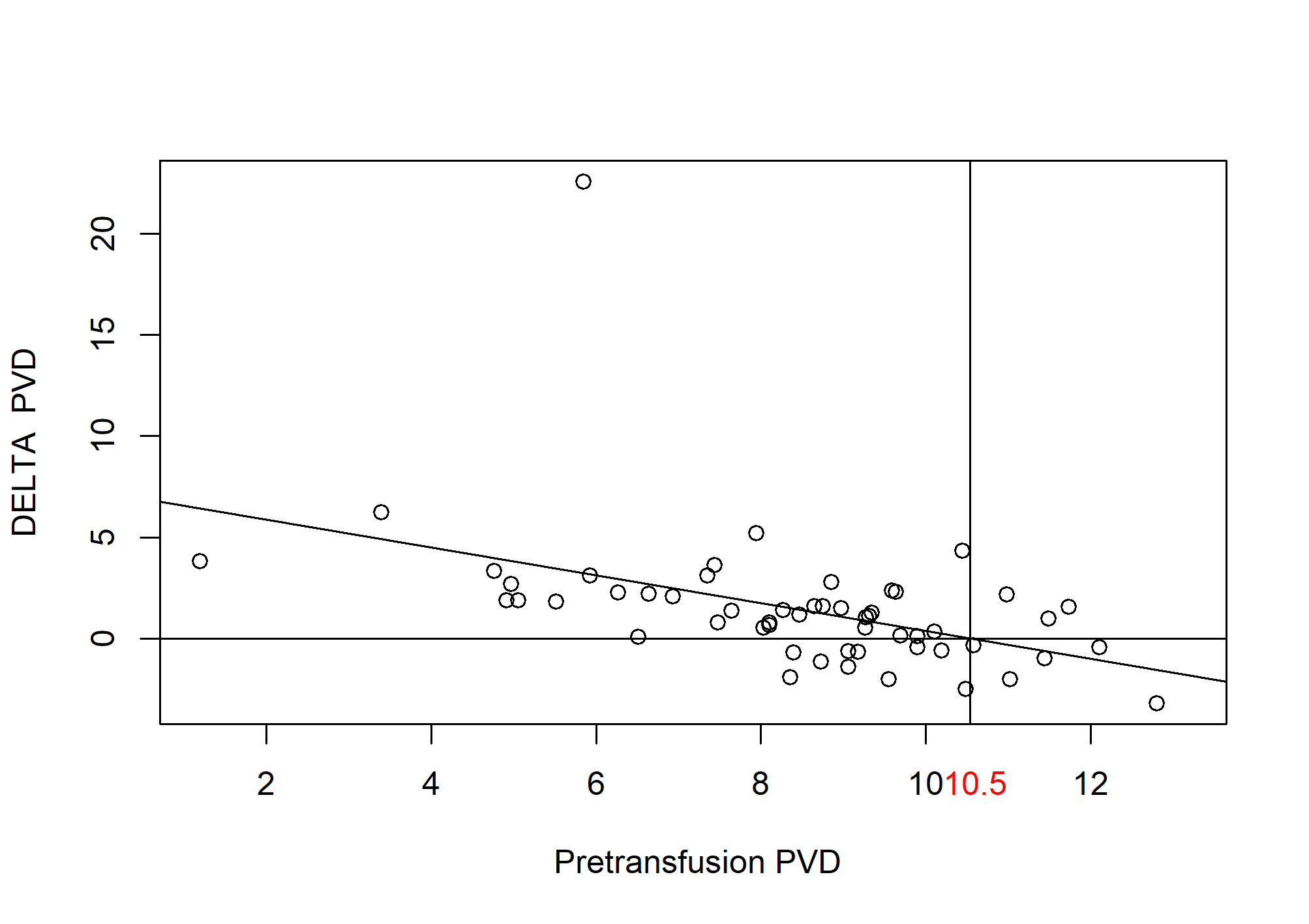


B

A

Supplement: Supplementary file 2 — Additional file 2: Figure S2. Δ TVD (A) and Δ PVD (B) after RBC transfusion in correlation with the pre-transfusion baseline. TVD = total vessel density; RBC = red blood cell; PVD = perfused vessel density. [file 13054_2020_2728_MOESM2_ESM.docx]
