# Supplementary material for: Sublingual microcirculation does not reflect red blood cell transfusion thresholds in the intensive care unit—a prospective observational study in the intensive care unit
Source: Crit Care. 2020 Jan 17;24:18. doi: 10.1186/s13054-020-2728-7 (PMC6969438; doi:10.1186/s13054-020-2728-7)
Supplement: Supplementary file 3 — Additional file 3: Table S1. Correlations of microvascular variables and hemodynamic variables. [file 13054_2020_2728_MOESM3_ESM.docx]

**Table S1** Correlations of microvascular variables and hemodynamic variables

| Delta | Δ MFI with  Δ MAP | Δ PPV with Δ MAP | ΔMFI with ΔSAP | Δ PPV with ΔSAP | Δ MFI with Δ DAP | Δ PPV with Δ DAP |
| --- | --- | --- | --- | --- | --- | --- |
| ρ | -0.266 | -0.263 | -0.132 | -0.107 | -0.045 | -0.118 |

*MFI* microvascular flow index; *MAP* mean arterial pressure; *PPV* proportion of perfused vessels;

*SAP* systolic arterial pressure; *DAP* diastolic arterial pressure
