# Supplementary material for: Sublingual microcirculation does not reflect red blood cell transfusion thresholds in the intensive care unit—a prospective observational study in the intensive care unit
Source: Crit Care. 2020 Jan 17;24:18. doi: 10.1186/s13054-020-2728-7 (PMC6969438; doi:10.1186/s13054-020-2728-7)
Supplement: Supplementary file 4 — Additional file 4: Table S2. Correlations of tissue oxygenation and pre-transfusion MFI/PPV. [file 13054_2020_2728_MOESM4_ESM.docx]

**Table S2** Correlations of tissue oxygenation and pre-transfusion MFI/PPV

|  | S_bt_O_2_ | S_df_O_2_ | S_tp_O2 |
| --- | --- | --- | --- |
| *p* for MFI | 0.199 | 0.319 | 0.007 |
| *p* for PPV | 0.294 | 0.397 | 0.072 |

*S_bt_O_2_* brain tissue oxygen saturation at the forehead; *S_df_O_2_* dorsum of the foot tissue oxygen saturation; *S_tp_O2* thenar prominence tissue oxygen saturation; *MFI* microvascular flow index;

*PPV* proportion of perfused vessels
